# Microbiomes *In Natura*: Importance of Invertebrates in Understanding the Natural Variety of Animal-Microbe Interactions

**DOI:** 10.1128/mSystems.00179-17

**Published:** 2018-03-13

**Authors:** Jillian M. Petersen, Jay Osvatic

**Affiliations:** a Department of Microbiology and Ecosystem Science, University of Vienna, Vienna, Austria

**Keywords:** microbiome, invertebrate, symbiosis, chemosynthesis

## Abstract

Animals evolved in a world teeming with microbes, which play pivotal roles in their health, development, and evolution. Although the overwhelming majority of living animals are invertebrates, the minority of “microbiome” studies focus on this group. Interest in invertebrate-microbe interactions is 2-fold—a range of immune components are conserved across almost all animal (including human) life, and their functional roles may be conserved. Thus, understanding cross talk between microbes and invertebrate animals can lead to insights of broader relevance. Invertebrates offer unique opportunities to “eavesdrop” on intricate host-microbe conversations because they tend to associate with fewer microbes. On the other hand, considering the vast diversity of form and function that has evolved in the invertebrates, they likely evolved an equally diverse range of ways to interact with beneficial microbes. We have investigated only a few of these interactions in detail; thus, there is still great potential for fundamentally new discoveries.

## Abstract (precis)

Animals evolved in a world teeming with microbes, which play pivotal roles in their health, development, and evolution. Although the overwhelming majority of living animals are invertebrates, the minority of “microbiome” studies focus on this group.

## PERSPECTIVE

Microbes evolved billions of years before animals; thus, all animals evolved among and with the teeming world of microbes in their environment. It should be self-evident that these microbes, with us since the dawn of our evolutionary history, have directed our evolution as much as any physical or chemical aspect of our environment. However, the “microbiome revolution” has only recently brought microbes’ beneficial roles widespread appreciation. In this Perspective, we define microbiome as the characteristic microbial community occupying the host-associated niche, according to Whipps et al. (1).

## EXCITING TIMES FOR MICROBIOLOGY AND SYMBIOSIS RESEARCH

**FIG 1:**
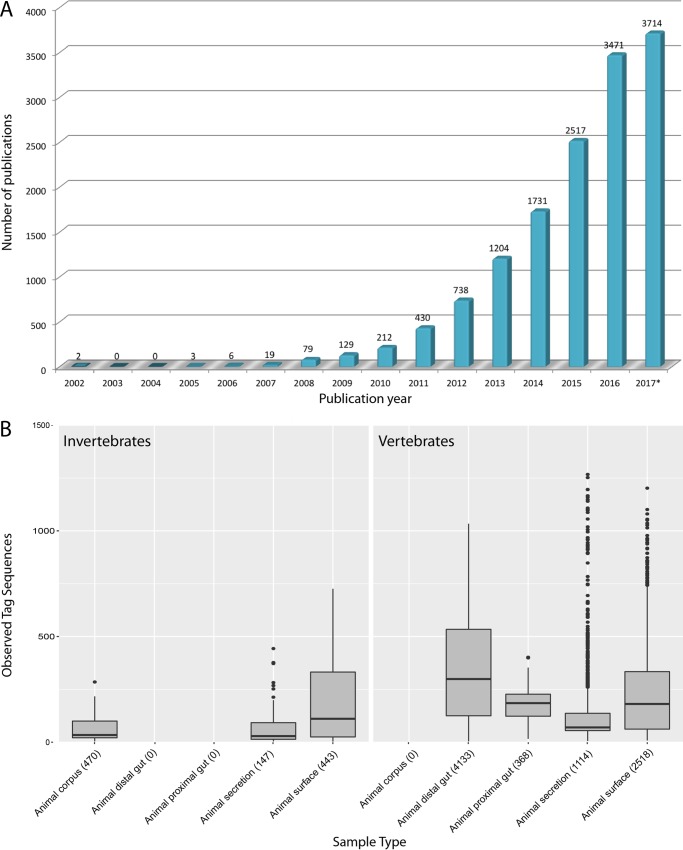
(A) The microbiome field has exploded in recent years. This graph shows the number of publications per year since the first appearance in 2002 of the term “microbiome.” (Source: ISI web of knowledge [https://webofknowledge.com/].) *, numbers for 2017 represent data from the period up to 4 December. (B) Invertebrate microbiomes are simpler than vertebrate microbiomes. These box plots show the numbers of unique tag sequences detected in samples of various categories. Categories were defined by the Earth Microbiome Project (http://www.earthmicrobiome.org/), which used a Deblur reference-free method of clustering sequences (15). In this data set of hundreds of samples, there is a clear trend toward simpler microbiomes in invertebrates containing fewer microbial sequences per individual. Intriguingly, the surfaces of vertebrate animals (right panel) and invertebrate animals (left panel) mostly appear to host similar numbers of different microbes. Despite the massive sampling effort of this extensive survey, there are differences in sampling methods and efforts between studies of vertebrates and invertebrates and a bias toward particular phylogenetic groups in both categories (see Fig. S2). Numbers in brackets indicate the number of samples in each category.

Life as we know it would not exist without the profound impact of beneficial host-microbe interactions. As Lynn Margulis, influential thinker and champion of the endosymbiosis theory for the origin of eukaryotes, elegantly remarked, “Life did not take over the globe by combat, but by networking” (2). There is now overwhelming evidence that life could not persist without the beneficial activities of microbes that underpin virtually every aspect of plant and animal biology, including human biology (3). The field of animal microbiome research, which aims to understand how microbes drive animal health, development, function, and evolution, has exploded in the past 5 years (Fig. 1). In a recent blog post, Kolter and Schaechter called this the “most exciting time in the history of microbiology” (4). This is clearly also an exciting time for symbiosis research, as beneficial microbes have never had a more prominent position in biology, medicine, and public interest. So far, we have investigated host-microbe interactions in detail in only a few (model) animals, and those studies have been grossly skewed toward vertebrates (see, e.g., Fig. S1 in the supplemental material). Thus, there is still enormous potential for discovering fundamentally new modes and mechanisms of interaction among the plethora of (non-model) animals and their microbial symbionts in nature.

10.1128/mSystems.00179-17.1 FIG S1  Distribution of all host-associated samples from the Earth Microbiome Project according to origin (invertebrate plant, unknown, vertebrate). Download FIG S1, PDF file, 0 MB. Copyright © 2018 Petersen and Osvatic. 2018 Petersen and Osvatic This content is distributed under the terms of the Creative Commons Attribution 4.0 International license.

10.1128/mSystems.00179-17.2 FIG S2  Distribution of the Earth Microbiome Project invertebrate-associated samples, according to scientific names of the host animals, as defined by the Earth Microbiome Project. Download FIG S2, PDF file, 0 MB. Copyright © 2018 Petersen and Osvatic. 2018 Petersen and Osvatic This content is distributed under the terms of the Creative Commons Attribution 4.0 International license.

Although most “microbiome” studies performed to date focused on vertebrates such as mice and humans, the vast majority of animal diversity is in the so-called “spineless majority,” the invertebrates. Their biodiversity is so extraordinary that, by some estimates, only 3% of animal species alive on Earth today are not invertebrates (5). The other 97% are insects, crabs, worms, clams, snails, comb jellies, corals, lobsters, sea urchins, spiders, and any of a range of other such life forms lacking a vertebral column. Despite their overwhelming dominance of the biosphere, we would wager that, if asked, a nonexpert would be able to name more vertebrate species than invertebrate. Humans and all other vertebrates share much of their basic biology with invertebrates. For example, both use the ancient innate immune system to interact with the microbes in their bodies and their environment. The innate immune system emerged early in metazoan evolution and is thus conserved across virtually all animal life. Its dysfunction is the cause of many human diseases; therefore, mechanisms of cross talk between microbes and the innate immune system are of broad interest and may be widely conserved.

## MICROBIOMES OF THE “SPINELESS MAJORITY”

Although they face an array of diverse microbes in their natural environments, a surprisingly large range of invertebrates have evolved exclusive associations with only one or a few microbial types. For example, of the 30 million insect species that possibly exist on Earth, 1 in 5 may harbor intracellular bacterial symbionts (6). Microbial symbionts and their invertebrate hosts associate faithfully over their lifetimes, across generations, and over evolution. Hosts and symbionts diverge and often reproduce, evolve, and speciate in concert. The microbes can form profuse “pure cultures” in or on the body of the host, reaching densities sometimes higher than *Escherichia coli* achieves in rich culture media (7). These observations raise two key questions. (i) How can these invertebrate animals, which lack the “memory” function of the antibody-based adaptive immune system, achieve this extraordinary specificity? (ii) How do they maintain such strict control over the growth and division of this massive population of microbes, which sometimes start off as a miniscule population of only a few cells?

Answers are beginning to emerge from a number of experimental host-microbe models. Binary associations, where one animal species hosts one prominent microbial symbiont species, have been a source of major breakthroughs in understanding the molecular basis of beneficial host-microbe interactions. For example, the association between marine bobtail squid and bioluminescent *Vibrio* bacteria, one of the best-known experimental models of symbiosis, has revealed the key roles of diverse components of the innate immune system in host-microbe communication and the profound influence of bacterial symbionts on the animal’s development and circadian rhythms (8, 9). Such intimate associations with specific microbes could leave evolutionary imprints on the host’s immune biology. However, with a few exceptions, the general effects of these prominent one-to-one symbioses on how these animals interact with other microbes, for example, those in their digestive tracts or on their outer surface, are still poorly understood, as is their influence on immune system evolution. One exception is the pea aphid *Acyrthosiphon pisum*, which shows evidence of immune system degeneration, possibly linked to its association with intracellular symbionts (10).

## THE UNIQUE ADVANTAGES OF INVERTEBRATE MODELS

Our ever-more-detailed picture of life’s inner molecular workings, a product of a century of work on model organisms, has logically come at the cost of a broad view of the range of biological solutions that have evolved in nature to allow animals and microbes to live a cooperative existence. Invertebrates have (at least) two outstanding features that make them ideal for understanding this variety. First, invertebrate microbiomes tend to be far simpler than vertebrate microbiomes. There has been some debate about this assertion, possibly because some invertebrates such as corals, sponges, and termites are known to host highly complex microbiomes, and also because systematic studies that generate truly comparable data are rare (11–14). We have reexamined this theory using data from the most extensive microbiome survey to date, the Earth Microbiome Project. These data are highly comparable thanks to the use of standard experimental and analysis methods (15). Our analysis confirms that microbiomes of invertebrates are generally simpler than those of vertebrates (Fig. 1). This means that fewer microbes associate with invertebrate individuals than with vertebrate individuals; thus, the molecular dialog between the host and individual members of its microbiome, a prerequisite to establishing and maintaining such specific and long-term partnerships, can be more easily deciphered in invertebrates.

Second, just as the invertebrates represent rich natural diversity, they have evolved virtually every known type of beneficial host-microbe interaction. The microbial symbionts can be passed strictly from parent to offspring (vertical transmission) or be taken up strictly from the environment during development (horizontal transmission) or participate in a mixture of the two (16). The association can be obligate for both host and microbe or optional (termed “facultative”) for either partner at certain stages of development. Invertebrates have also evolved a multitude of solutions to the problem of housing microbial symbionts. The symbionts can be hosted outside or inside the body. If they are inside, they can be found outside host cells or, in a uniquely intimate form of symbiosis, inside cells exclusively dedicated to housing symbionts. So far, all known examples of intracellular symbioses except one have been found in invertebrates (17). In summary, for virtually every conceptual issue concerning host-microbe research, there is an invertebrate in which it could be investigated.

Paradoxically, intracellular associations are not always obligate for both partners despite their advanced level of cellular integration. There are many examples of marine invertebrate animals with intracellular symbionts that are taken up from the environment during development. Many of these are chemosynthetic symbioses, where symbiotic chemosynthetic bacteria transform reduced, sometimes toxic chemicals in the environment into a rich source of nutrition for their hosts (18). It is often assumed that these symbionts have active, free-living forms. For some animals such as the deep-sea *Riftia* tubeworms, symbionts were recently shown to escape dead hosts to seed the environment with presumably active free-living forms (19). For others, such as *Bathymodiolus* mussels, the symbionts likely disperse in a dormant or inactive state because they have lost central metabolic enzymes (20). Intracellular symbioses are of major evolutionary significance—the remnants of ancient alphaproteobacterial invaders exist today as mitochondria in virtually every animal cell (21). Intracellular bacteria were long thought to be invisible to the immune system, but discoveries such as that of the expression of specific receptors for microbial components in animal cell nuclei are calling this assumption into question (22). In addition, when these symbionts are taken up from the environment, their journey into host cells must bring them in contact with the host’s immune system; however, there is so far no experimental system in which beneficial intracellular bacterial symbionts infect an animal host from the environment where this infection process could be studied.

**FIG 2:**
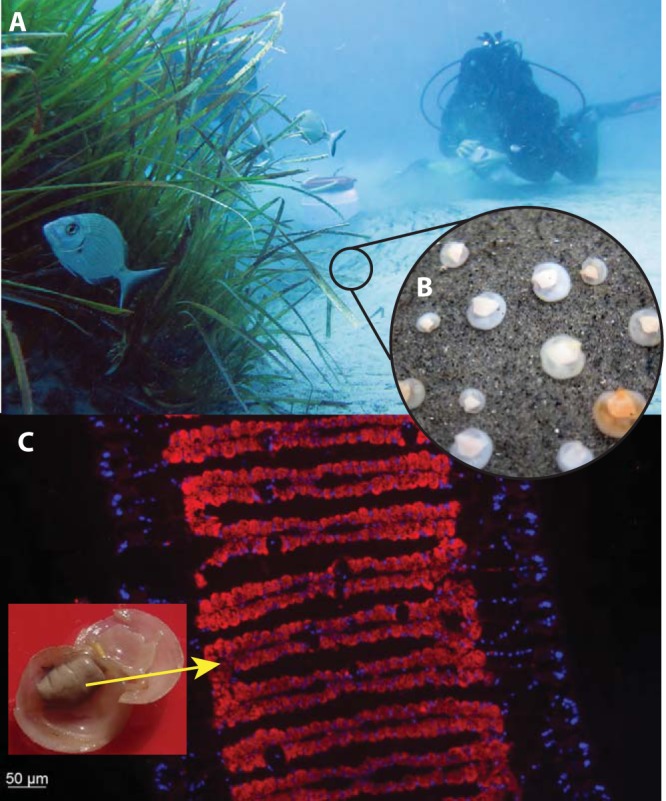
Some marine invertebrates such as lucinid clams host a massive population consisting of an almost pure culture of bacteria in their tissues. (A) The natural habitat of the clams around seagrass beds in the Mediterranean Sea (photo courtesy of Ulisse Cardini). (B) These clams (about 1 cm in length) have an almost transparent shell through which the symbiont-hosting organ, the gill, can be clearly seen (photo courtesy of Ulisse Cardini). (C) Fluorescence in situ hybridization (FISH) performed with probes specific for the symbionts showed that epithelial cells of the gill filaments are packed with symbiotic bacteria (insert photo courtesy of Marc Mussmann; FISH image courtesy of Anna Kemper).

Intracellular chemosynthetic symbioses offer unique opportunities for understanding the molecular underpinnings of beneficial host-microbe interactions. Like those in the squid-*Vibrio* model, their microbiomes are naturally very simple—most hosts associate with one or only a few symbiont species. Recent breakthroughs in cultivating the symbionts promise a new era in understanding how these intimate and sometimes ancient symbioses are established (23). Some of the host animals can also be cultivated and experimentally manipulated in the laboratory. For example, lucinid clams were raised aposymbiotically several years before *Euprymna scolopes*, and yet their reciprocal molecular interactions upon “first contact” are still completely unknown (24) (Fig. 2). In addition, symbiont loss can be experimentally induced, by depriving adult lucinids of their symbionts’ energy sources. The symbiosis can be restored by returning the clams to their native habitat, where free-living symbionts colonize the sediments (25). The symbiosis can also be restored in the laboratory by adding symbiont cells harvested from freshly collected adults. Surprisingly, recent research suggests that these essentially aposymbiotic adults can reestablish symbiosis only with the symbiont strain with which they had previously associated before symbiont loss and that highly similar (but not identical) strains fail to colonize these hosts (26). This represents a striking contrast to the behavior of the juveniles, which are competent to establish symbiosis with any one of a range of different symbiont strains (27). These experiments show tantalizing indications of shifts in symbiosis flexibility occurring during animal development which mirror the early development of human microbiomes (28). They also raise the sensational idea that these invertebrate animals might have a specific immune “memory” function, which would throw into question our current understanding of the function and specificity of the innate immune system.

Nobel laureate and biochemist Jacques Monod famously quipped that “anything true of *E. coli* must also be true of elephants,” but when it comes to the microbiome, even within a single species, each individual can host its own unique microbial ecosystem (29, 30). Individual differences matter. For example, the field of human medicine is waking up to the importance of interpersonal differences, which cause many drugs to be highly effective in some patients and ineffective or even harmful in others (31). These differences are thought to be due to diversity in the underlying molecular causes of disease and to the influence of each person’s diverse and yet unique set of microbial partners (32). In the future, we will likely discover that many of the mechanisms at work in host-microbe interactions are widely conserved across vast phylogenetic and evolutionary spaces. However, the magnificent diversity of modes of host-microbe interactions in the invertebrates demonstrates that major differences in the underlying mechanisms, even in closely related organisms, are likely. We now have a range of “omics” technologies to investigate non-model (symbiotic) organisms in remarkable molecular detail, even if methods for genetic manipulation are not always available. Embracing the idea of the range of diverse host-microbe associations in nature will lead to a much better understanding of the varied mechanisms by which microbes drive animal health, development, and evolution.
