# Supplementary figures and images for: Microbiomes In Natura: Importance of Invertebrates in Understanding the Natural Variety of Animal-Microbe Interactions
Source: mSystems. 2018 Mar 13;3(2):e00179-17. doi: 10.1128/mSystems.00179-17 (PMC5850079; doi:10.1128/mSystems.00179-17)

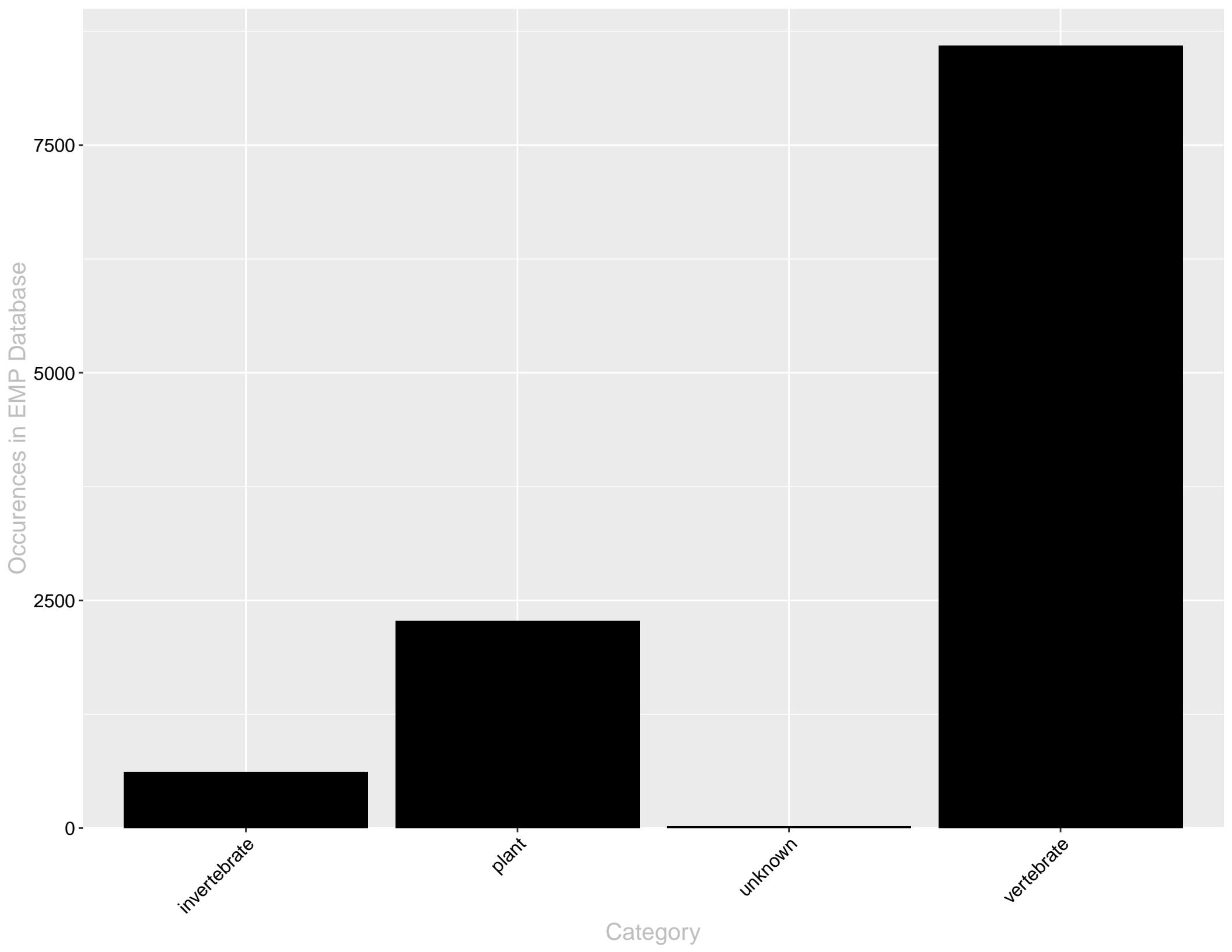

Supplement: FIG S1 [file sys002182190sf1.pdf]

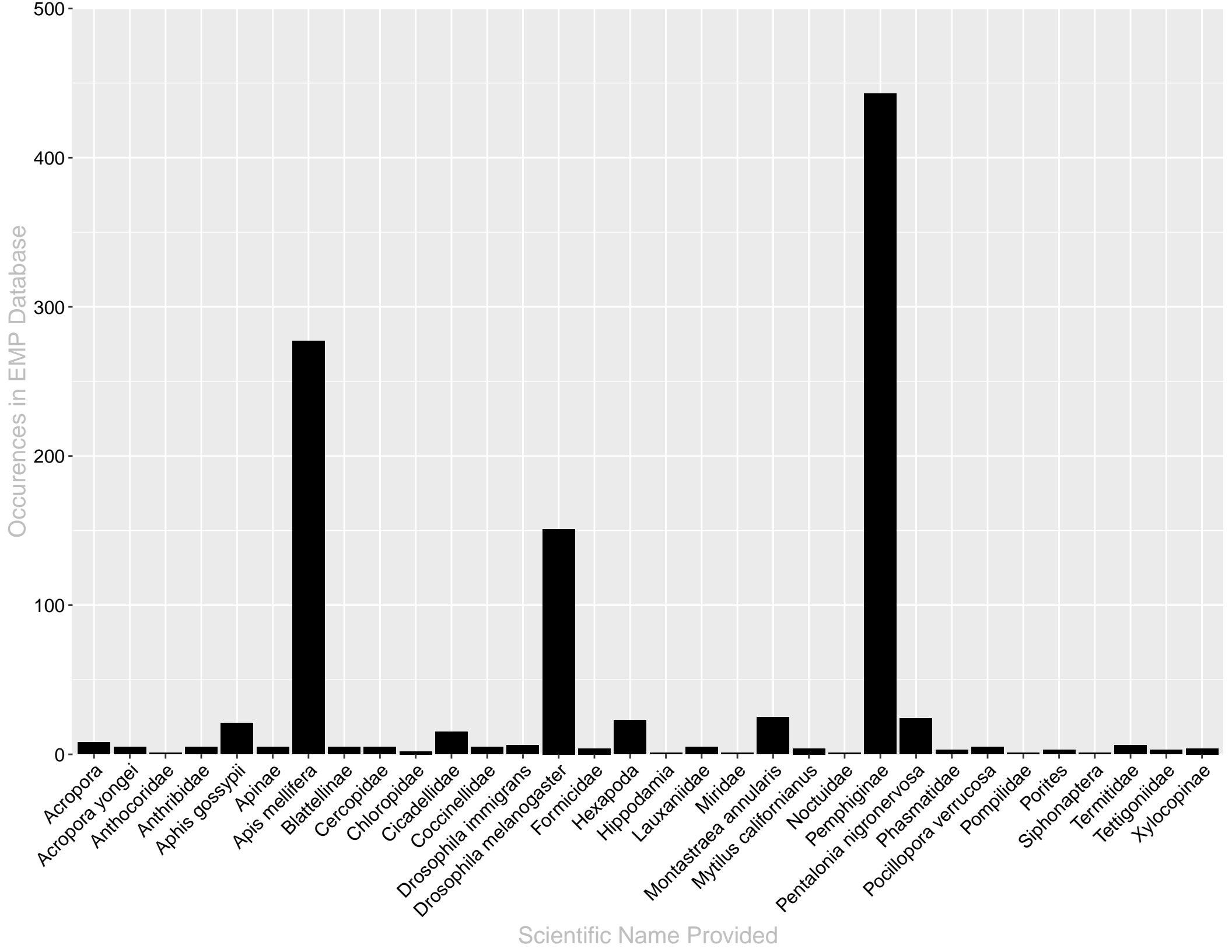

Supplement: FIG S2 [file sys002182190sf2.pdf]
